# Draft Genome Sequence of *Bacillus* sp. Strain NSP9.1, a Moderately Halophilic Bacterium Isolated from the Salt Marsh of the Great Rann of Kutch, India

**DOI:** 10.1128/genomeA.00835-13

**Published:** 2013-10-10

**Authors:** Rinku Dey, Kamal Krishna Pal, Dharmesh Sherathia, Trupti Dalsania, Kinjal Savsani, Ilaxi Patel, Manesh Thomas, Sucheta Ghorai, Sejal Vanpariya, Rupal Rupapara, Priya Rawal, Bhoomika Sukhadiya, Mona Mandaliya, Anil Kumar Saxena

**Affiliations:** Microbiology Section, Directorate of Groundnut Research, Junagadh, Gujarat, Indiaa; Division of Microbiology, Indian Agricultural Research Institute, New Delhi, Indiab

## Abstract

We report the 4.52-Mbp draft genome sequence of *Bacillus* sp. strain NSP9.1, a moderately halophilic bacterium isolated from the salt marsh of the Great Rann of Kutch, India. Analysis of the genome of this organism will lead to a better understanding of the genes and metabolic pathways involved in imparting osmotolerance.

## GENOME ANNOUNCEMENT

*Bacillus* sp. strain NSP9.1 (16S rRNA GenBank accession number JF802181), a moderately halophilic and endospore-forming bacterium, was isolated from the salt marsh of the Great Rann of Kutch, India. It grows optimally at a 7.5% NaCl (range, 0 to 15%) concentration in medium at 37°C and pH 7.5. The genome of *Bacillus* sp. NSP9.1 was sequenced with a view to understanding the mechanism(s) of osmotolerance and mining the relevant gene(s).

The whole genome of NSP9.1 was sequenced using the Roche 454 genome sequencer (GS FLX) at Macrogen Inc., South Korea, through Sequencher Tech Pvt. Ltd., Ahmedabad, India, by both shotgun and mate-paired library sequencing. In shotgun sequencing, an average read length of 412 bp was obtained from 760,964 reads of 314,016,162 bases. Sequencing of mate-pair libraries generated 148,132 reads of 70,321,948 bp and 148,351 reads of 70,918,411 bp, with average read lengths of 474 bp and 478 bp, respectively.

GS De Novo Assembler v 2.6 (1) was used for assembling the reads. The genome assembly of *Bacillus* sp. NSP9.1 (G+C content of 45.64%) has approximately 98-fold coverage. It contains 8 scaffolds totaling 4,520,573 bp, with an average length of 565,071 bp (largest, 2,378,181 bp, and smallest, 4,984 bp). The scaffolds consist of 33 contigs totaling 4,511,959 bp, with an average length of 136,726 bp. *N*_50_ scaffold lengths of 2,378,181 bp and *N*_50_ contig lengths of 219,915 bp (smallest, 1,618 bp, and largest, 874,414 bp) were obtained. All assembly data were deposited in the DDBJ/EMBL/GenBank nucleotide sequence database.

The draft genome sequence of *Bacillus* sp. NSP9.1 was annotated by using the RAST server (2), Glimmer 3 (3, 4), GeneMark (5, 6), the KEGG database (7), tRNAScan-SE (8), RNAmmer (9), and Signal P4.1 (10) for predicting subsystems, coding sequences (CDS), tRNA and rRNA genes, signal peptides, pathways involved, etc.

Using the different software tools, we predicted 4,860 CDS, with 3,950,907 bp in the CDS. There were 144 RNA-encoding genes (140 tRNA and 4 rRNA genes) and 468 subsystems. Among the CDS, 2,775 are not in a subsystem (nonhypothetical, 1,088; hypothetical, 1,687) whereas 2,085 CDS (nonhypothetical, 1,948; hypothetical, 137) are in a subsystem. RAST annotation also revealed 101 genes associated with stress responses, including 14 associated with osmotic stress (osmoregulation, 1; choline and betaine uptake and betaine biosynthesis, 13), 47 with oxidative stress (protection from reactive oxygen species [ROS], 8; oxidative stress, 28; NADPH:quinone oxidoreductase 2 reactions, 1; glutathione:nonredox reactions, 1; redox-dependent regulation of nucleus processes, 6; glutaredoxins, 3), 1 with cold shock, 16 with heat shock, 1 with detoxification, 21 with no subcategory, and 1 with the periplasmic stress response. Use of the Signal P4.1 server predicted 436 signal peptides. A total of 2,226 open reading frames (ORFs) were mapped to different biochemical pathways of KEGG (K00003 to K16706). A number of genes for ABC transporters (map02010) have also been mapped, including genes associated with osmoprotectants and two-component systems (map02020), such as those for response to K^+^ limitation and K transport and salt stress degradative enzymes, etc.

We are further exploring the genome of *Bacillus* sp. NSP9.1 in our laboratory to understand the mechanisms of salinity tolerance and to determine the genes, biochemical pathways, and metabolites involved in osmotolerance.

### Nucleotide sequence accession numbers.

This whole-genome shotgun (WGS) project has been deposited at DDBJ/EMBL/GenBank under the accession number AUQZ00000000. The version described in this paper is version AUQZ01000000.
